# Air-conducted ocular vestibular-evoked potential

**DOI:** 10.6026/973206300212557

**Published:** 2025-08-31

**Authors:** Saumya Pandey, Sangeeta Gupta, Ramashankar Rath, Gaurav Gupta

**Affiliations:** 1 Department of Physiology, All India Institute of Medical Sciences (AIIMS) Gorakhpur (Uttar Pradesh), India; 2 Department of Community medicine and Family medicine, All India Institute of Medical Sciences (AIIMS) Gorakhpur (Uttar Pradesh), India; 3 Department of General Surgery, All India Institute of Medical Sciences (AIIMS), Gorakhpur, (Uttar Pradesh), India

**Keywords:** VEMP, O-VEMP, vestibular, inferior oblique muscle, tone burst stimuli, click stimuli, otolith organs, latencies, amplitudes and threshold

## Abstract

Ocular vestibular-evoked myogenic potential (O-VEMP) test can have great clinical value if potential influences of stimulus characteristics are known. Therefore, it is of interest to examine the effects of different variables on O-VEMP responses by delivering air-conducted sound stimuli in the form of tone bursts and clicks in sitting and supine positions in healthy participants. Total 60 healthy participants in the age range of 18-60 years were investigated and statistical analysis was performed to find the variations in O-VEMP responses (p-value of <0.05 was considered as significant). Tone burst stimuli resulted in larger amplitudes (p=0.005, p = 0.0008) and lower thresholds (p = 0.005, p=0.03), for right and left ear respectively, while supine position produced larger O-VEMP amplitudes as compared to those in sitting position (p<0.001). Potential influence of stimuli, recording conditions, age and gender on O-VEMP response should be borne in mind during clinical interpretation.

## Background:

Vestibular evoked myogenic potentials (VEMPs) have evolved into fundamental and increasingly recognised methods in the neuro-otology assessment set. Recent technological improvements have enabled clinicians to evaluate otolith function using VEMP testing [1, 2]. The short latency, vestibular-dependent reflexes are elicited by stimulating the ears using air-conducted sound or skull vibration. These reflexes are then measured using surface electrodes placed across the neck, over sternocleidomastoid (SCM) muscles (cervical VEMPs) or beneath the eyes (ocular VEMPs) [2]. C-VEMP (cervical VEMP) has become a well-established clinical test of vestibular function [3]. However, recording VEMP from inferior extra ocular muscles of the eye has been the area of interest of recent research. The neuro-otologic test regimen has, hence, been lately expanded to include ocular vestibular-evoked myogenic potentials (O-VEMPs) as a novel measure of the vestibulo-ocular reflex [4]. O-VEMPs are documented in the presence of ocular myogenic responses. In contrast to the c-VEMP, which examines the ipsilateral descending vestibular route, the O-VEMP has been successfully used to assess the ascending vestibular pathway through the vestibulo-ocular reflex system [5]. Ocular vestibular-evoked myogenic potential (O-VEMP) are excitatory electromyographic (EMG) response measured over the inferior oblique (IO) muscle. They assess contralateral (to recording electrodes/IO muscle) utricular macula and superior vestibular nerve functions [6]. Researches indicate that O-VEMP is produced by otolith afferents in the superior vestibular nerve that contains all utricular afferents but a limited number of afferents from the anterior saccule [6]. Therefore O-VEMP is clinically employed to evaluate the utricle's function [7]. Winters *et al.* investigated the O-VEMP changes with respect to frequency of the air-conducted stimuli in patients with Ménière's disease [8]. Though O-VEMP can be used like other vestibular function tests, to aid diagnosis or monitoring the disease progression/recovery, yet the test has an advantage over the existing tests of otolith function as the reflex remains abnormal even after central compensation has occurred and is more practical [9]. O-VEMP parameters have been reported to have excellent reliability owing to several factors [10]. Sustained gaze deviation required for the O-VEMP test produces little measurable muscle fatigue [11]. The excellent reliability have also been attributed to several other factors including less room for error in optimal electrode placement, relatively small background noise of extraocular muscle activation etc. On the other hand, the c-VEMP response has relatively noisy background of SCM contraction [10]. Welgampola *et al.* reported that patients with SCDS (superior canal dehiscence syndrome) had significantly elevated tone-evoked O-VEMP amplitudes at 120 dB SPL peak intensity, suggesting that the O-VEMP amplitude in response to a single standard stimulus intensity level provides useful diagnostic information [3]. On the other hand, the evaluation of SCDS patients with the threshold c-VEMP test, multiple test runs for each ear are required, and thus, prolonged SCM contraction must be maintained for multiple times. Hence, the testing sessions would be shortened if O-VEMP amplitudes used for SCDS diagnosis. O-VEMPs have been suggested to be a better technique for gauging the effects of therapy, disease progression, or aging than the c-VEMPs [10].

O-VEMP stimulus characteristics are known to influence the response prevalence, amplitude and latency of the records. There is a significant variability in individual responses to stimuli of different shape and frequency and there is not one best VEMP stimulus [12, 13]. A few researchers in the past have documented the effect of different stimuli, including air-conducted tone burst, air-conducted clicks, bone conducted 500 Hz tone bursts, mechanical head taps and electrical stimulation at the mastoid, on O-VEMP attributes [3, 4- 5, 11]. Notwithstanding, the above evidences include data from modest number of participants and represent great variability in data collection methods. Comparatively evaluating these studies has been difficult. A relatively large age-stratified population has very sparsely been studied for O-VEMP response characteristics. Moreover, the body of information pertaining to the influence of various stimuli on the elicitation of the c-VEMP response has been extensive. On the contrary, there exists a paucity of knowledge regarding the impact of various stimuli on the O-VEMP response [14, 15]. Owing to the potential impacts of age and stimulus parameters on O-VEMP amplitudes, latencies and threshold values, patients should ideally be compared to normal age-matched controls tested under the same stimulus and recording conditions [16]. Therefore, it is of interest to obtain a standardised data with respect to the variables known to influence the O-VEMP parameters in the healthy study participants for optimising the clinical applicability of this relatively novel vestibular function test.

## Materials and Methods:

This analytical cross-sectional study involving 60 subjects was conducted during a period of April 2023 to March 2024. Since this was an exploratory study, to satisfy the central limit theorem, a total of 30 males and 30 female participants were included by convenience sampling. The study protocol was approved by the Institute's Human Ethics Committee (IHEC ref no: IHEC/AIIMS-GKP/BMR/123/2023). Subjects with the age-range of 18-60 years, with normal otological and vestibular examination were included in the study. Exclusion criteria for the study participants were those with the history of otological, vestibular, neurological or neuromuscular disorders or those with history of cerebral trauma. The participants studied were healthy attendants of the patients visiting hospital out-patient department (OPD) and Neurophysiology Laboratory, AIIMS Gorakhpur. The participants were given a detailed explanation of the duration, kind, and goal of the study, and each participant provided informed written consent.

## Procedure:

## Recording of ocular vestibular evoked myogenic potential (O-VEMP):

O-VEMP test was performed on Neuro-MEP 8 (8-channel NCS, EMG and multi-modality EP system) with Neuro-MEP.netω electromyography software (M/S Neurosoft Ltd, Ivanovo, Russia) at Neurophysiology Laboratory, AIIMS Gorakhpur. O-VEMP was recorded by a single channel recording in a quiet environment and at a uniform temperature. Information regarding the procedures to be followed was given to the participants prior to the test. Subjects were instructed to perform the appropriate movements for recording VEMP. Pre-stimulus EMG was recorded for 20 ms. The pre-stimulus EMG was useful to measure the level of background noise in the recording, from which the response peaks will be detected. Reliable VEMPs were identified as those with consistently exceeding the residual background EMG seen in the pre-stimulus trace. Participants received both tone burst and click stimuli in different sets. The active electrode was positioned on the face just inferior to the eye, around 1 cm below the center of the contralateral lower eyelid, the reference electrode about 1.5 cm below the active one [5, 17, 18]. Ground electrode was placed on the forehead. Electrode impedance was maintained below 5 kiloohms. The 500 Hz tone burst (rise/fall time 0 ms, plateau 2.67 ms, stimulation rate 5/s) and click acoustic stimuli (0.1 ms duration) were used. O-VEMP stimuli were delivered monaurally at 95 dB nHL with rarefaction polarity, using TA-01 headphones (Table 1 - see PDF)

## Subject positioning and instructions for O-VEMP:

Ocular VEMP recording was performed separately for right and the left ear and in the following different body positions:

[1] The participants were first made to sit in the upright position with head level. They were instructed to hold their gaze at a target approximately 20° above the horizontal.

[2] Lying supine with chin tilted down and eyes elevated 20° to a target on ceiling.

Recordings for O-VEMP were taken in both sitting and supine positions under above mentioned electrode montages and stimulus settings. When responses were absent, the stimulus was repeated using maximum up-gaze for a single recording [19]. 250 sweeps of the stimuli were presented. The recording was repeated to check the replica of the peaks. Recordings from both the ears were obtained. O-VEMP was recorded with the different sets of experiments in each participant (Table 2 - see PDF). All the O-VEMP parameters were recorded, and analysis was performed for finding the effect of variation of stimulus and recording conditions on O-VEMP responses.

## O-VEMP variables:

[1] Latency: n1 and p1 (n10 and p16) (measured at the response peak)

[2] Threshold stimulus (the lowest amplitude sound stimulus that still elicits a reproducible O-VEMP response).

[3] Threshold asymmetry (interaural)

[4] Amplitude (peak to peak): n1-p1 (n10-p16)

[5] Amplitude asymmetry: The interaural asymmetry ratio (IAR) was calculated using the Jongkees' formula (right - left)/(right + left)

[6] Amplitude Asymmetry Ratio (AAR) = (AR-AL) / (AR+AL) x100

AR: amplitude of VEMP (with acoustic stimulus delivered to right ear)

AL: amplitude of VEMP (with acoustic stimulus delivered to left ear)

## Results and Discussion:

Out of 60 participants who underwent O-VEMP recording, 30 (50%) were males and 30 (50%) were females. These participants (n=60) were divided into two age groups, 18 to 40 years and 41 to 60 years. O-VEMPs were bilaterally present in all the participants in the age group with 18-40 years old subjects while in that with 41-60 years old subjects, O-VEMP responses were bilaterally absent in 6 participants. Hence, the overall response rates for O-VEMP was 90 % for both tone-burst as well as click stimuli. Previous researches have reported the response prevalence of O-VEMP which is mostly tone-burst evoked. Response rates of 90 % (for 500 Hz tone burst evoked O-VEMP) conform well to previous similar researches [6, 16]. Rosengren *et al.* (2011), reported response rates of 81%, 59%, and 65% of ears by AC tone bursts, clicks and bone-conducted (BC) tone bursts, respectively [20]. The analyses for studying the variation in O-VEMP measures with the type of stimulus (air-conducted tone burst and click) were performed with the standard recording settings (active electrode placed beneath the lower eyelid and the position of the subject as sitting) during the procedure. The mean, median, SD and IQR values of O-VEMP parameters and their comparison with stimulus variations (tone burst vs click stimuli) in total participants (n=54) as well as age-stratified analyses are shown in Table 3. When the O-VEMP parameters were compared between the groups with stimulus variation (AC tone burst and click), no significant differences were observed for n10 latency in both the ears, left ear p16 latency (total and in ≤40 years old subjects), amplitude asymmetry and threshold asymmetry (p > 0.05) (paired t-test) (Table 3 - see PDF). However, right ear p16 latency was found to be reduced with click-evoked O-VEMP with statistical significance when compared in total subjects along with reduced left ear p16 latency (in older participants) (p=0.04 and p=0.046 respectively) (paired t-test) (Table 3 - see PDF). Other previous studies also reported that click stimuli produced significantly shorter n1 and p1 latencies compared to 500 Hz tone bursts [5, 21]. The longer latencies produced by tone burst stimuli have been attributed to different excitation patterns of vestibular neurons. It has been reported that primary vestibular neurons respond to tone burst stimulus by double or triple firing. Hence, the longer latency associated with 500 Hz tone burst stimulus may be due to the influence of second or third electrical impulse "spikes" [22]. The role of greater rise/fall time of the tone-burst stimulus has also been implicated in the prolongation of the O-VEMP latencies (similar to c-VEMP latencies) [23]. The present study, albeit, suggests using tone burst stimulation for evoking better O-VEMP response.

On the contrary, n10-p16 amplitude for right ear (p = 0.005), that for left ear (p = 0.0008) (paired t-test), right ear threshold (p = 0.0075) and left ear threshold (p = 0.03) revealed significant difference between tone burst and click stimuli (Wilcoxon signed rank test) (Figure 1a-d - see PDF). Age-stratified analysis, however, revealed lack of such statistical significance for left ear n10-p16 amplitude (p=0.32), right and left ear threshold (p=0.42, 0.71, respectively) for the older participants (41-60 years) (Table 3 - see PDF). Click stimulus have a shorter duration (0.1 ms) while the stimulus duration for the tone burst are longer. Increasing stimulus duration typically increases VEMP amplitude, as the total sound energy delivered to the inner ear is increased (but up to a certain limit) [24, 25]. Hence, lower mechanical energy of click stimulus might be a reason for its reduced amplitude and increased threshold response [22]. Previous studies also reported largest amplitudes and lowest thresholds at 500 Hz tone burst stimulation when compared to click stimulation [12, 26 and 27]. On comparing sitting vs supine recording position, no significant differences were observed for n10, p16 latency for both right and left ears, amplitude asymmetry, thresholds and threshold asymmetry (p > 0.05) (paired t test). However, right ear amplitude (p = 0.000) and left ear amplitude (p = 0.0001) (Wilcoxon signed rank test) showed significant differences between the sitting and supine recording position (Table 4 - see PDF, Figure 2a, b - see PDF). Supine position produced larger O-VEMP amplitudes than those in sitting position. Age-stratified analysis also revealed similar results (Table 4 - see PDF). A previous study reported that sitting position produced the shortest n1 latency, but no significant changes in the amplitude and threshold were reported [28]. Another similar study compared the effect of four different testing positions (sitting position, supine position, lying on right side and lying on left side) on O-VEMP parameters and reported that supine positions elicited the most O-VEMP responses in the participants, but sitting upright was found to be the optimum position for best threshold [29].

The findings from our research, in conjunction with the findings from the studies in the past stated above, favours the fact that O-VEMP recording should be done in the supine position given that there are no O-VEMP responses observed while the subject is sitting upright. O-VEMP responses were compared between the age-groups under standard stimulus and recording conditions (air-conducted tone burst stimulation with patient seated upright). Response prevalence was 100 % in 18-40 years old subjects while 90 % in the older participants. A previous study reported that half of the healthy individuals aged 40 years old and above had absent O-VEMPs [16]. The commonly reported decrease in the response rates and the O-VEMP amplitudes with age have been widely explained by well-documented neuroanatomic age-related changes that occur in the peripheral vestibular system. It has been stated there was age-related loss of 3% per decade in individuals above 40 years of age from the vestibular nucleus complex. In the age range of 40 to 90 years a 6% per decade vestibular epithelium hair cell loss was observed by Rosenhall [30]. Thus, it may be inferred that the deterioration that occurs with advancing age impacts the pathways responsible for regulating the vestibular reflexes at various levels. No significant difference between the age groups was observed for right and left ear n10 and p16 latencies (p > 0.05) (unpaired t-test). The findings are in line with the previous studies [10, 31]. Few studies which have reported latency prolongation with age, differ with the present research not only in sample size and age-range of the subjects but also methodologically such as in electrode placement, stimulation mode (bone-conducted vibration) etc. Interestingly, a possible gender effect has been implicated, stating that the increase in the latencies was only significant in men, in few studies [32]. The present study also found increased n10 and p16 latencies in the male participants (n=27) as compared to the female participants (n=27) (p<0.05). This prolongation might hence be attributed to the aforementioned possibility of more pronounced aging effects in men (Table 6 - see PDF). In line with the above implications, the absence of any statistically significant prolongation of O-VEMP latencies in males, in a study by Sung *et al.* (2011) can be explained by the younger participants (age-range : 24-33 years) included in the study [33].

Right ear and left ear n10-p16 amplitudes significantly reduced in the older age group (p = 0.00005 and p = 0.0000 respectively) (Mann Whitney U test). The results exhibiting a decrease in the amplitude with increasing age in our study conform to the studies in the past which have also reported that the decrease in O-VEMP amplitude has been independent of the stimulus used [10, 20, 31 and 32]. The above-mentioned fact might explain the diminished amplitudes [2.8 (1.22) and 2.6 (1.08)] [median (IQR)] obtained for both the type of stimulus delivered and no statistical difference obtained (p=0.32) (left ear) in the individuals above 40 years age (Table 3 - see PDF). Morphological alterations in the otolithic organs and corresponding changes in the neural function may be the attribute factors. Also, these outcomes might be due to age-dependent neuronal deterioration of the vestibulo-ocular reflex. Another possible reason could be a reduction in the number of myelinated primary vestibular afferents taking place above 40 years [34]. Also, right and left ear threshold increased with significant differences between the two groups (p = 0.0086, p = 0.0006 respectively) (unpaired t-test) (Table 5 - see PDF). Increase in the threshold in the older subject's concord with the previous reports [35, 36]. This can explain the increased thresholds for both tone-burst as well as click stimulus and hence lack of any statistically significant differences in the older participants (p=0.42 and 0.71 for right and left ear threshold comparison among the two stimuli respectively) (Table 3 - see PDF). Pearson and Spearman correlation test were used to analyze the correlation of O-VEMP parameters with age. The Spearman correlation analysis revealed a significant negative correlation between the age and n10-p16 amplitude in both the ears [right ear (r = -0.5102, p = 0.0000) and the left ear (r = -0.5385, p = 0.0000)]. The findings, hence, implied that as the age increased, the amplitude of O-VEMP decreased (Figure 3a - see PDF & b - see PDF). O-VEMP responses were compared between males and females under standard stimulus and recording conditions (air-conducted tone burst stimulation and with subjects seated upright). The mean, median, SD and IQR values of O-VEMP parameters and their comparison between males and females are shown in Table 6 (see PDF). No significant differences were observed for threshold (unpaired t-test). O-VEMP amplitudes were found to be increased in males but the statistical significance was not attained in our study (p>0.05) (Mann Whitney U test) (Table 6 - see PDF). Sung PH *et al.* (2011) demonstrated that mean O-VEMP amplitude in males was significantly larger than that of females, regardless of the mode used (air-conducted or bone-conducted stimulation). They reported significant correlations between the BMI and O-VEMP amplitude and attributed the increase in amplitude in males to variance in the muscle bulk between males and females [33]. We observed increased n10 and p16 latencies in the male subjects with statistical significance (Table 6 - see PDF) (p<0.05) (unpaired t test). Gender differences in O-VEMP parameters have not been abundantly explored in the past. Erbek *et al.* (2011), investigated the same, yet could not obtain significant gender influence on O-VEMP results [37]. Another similar study also reported no significant gender differences in latencies or peak-to-peak amplitude for O-VEMPs [31]. Based on the findings of the present study along with the previous similar literature, the O-VEMP results owing to the gender differences require further evidences. Some aging effects however have definitely been implicated to explain prolonged latencies in males, in few researches. The current study found upper limit of amplitude asymmetry for tone burst-evoked O-VEMP responses (maximum percentage of amplitude asymmetry) to be -25.84% and that for clicks to be -26.97% (sign convention is attributable to Jongkees' formula). Piker *et al.* have defined the upper limit of tone-burst-evoked O-VEMP asymmetry as 34 % which is slightly higher than our findings [38].

## Limitations:

Older participants over 60 years old were not included in this study. Consequently, the impact of aging on VEMP responses could not be assessed in this older age cohort. In our study, bone-conducted (BC) stimulation was not employed to elicit VEMP responses. BC stimulation has been reportedly found to yield O-VEMP responses, where bilateral responses are absent, and among the elderly population. With regard to the superior gaze angle (for O-VEMP recording), a laser pointer embedded in the forehead module have been used by some researchers to control the angle of gaze. This has reported to allow a consistent amount of superior gaze. However, we used the conventional method (marking a static visual target on the ceiling).

## Conclusion:

Tone-burst-evoked stimuli (500 Hz) produced larger O-VEMP responses than those with clicks, though with fairly similar response rates in normal healthy adults (≤60 years). Potential absence of air-conducted tone burst/click O-VEMP responses in older individuals and increased O-VEMP latencies in males warrant the importance of inclusion of age and gender in the evaluation norms in addition to stimulus types and recording conditions.

## Funding:

None

## Ethical approval:

The study protocol was approved by the Institute's Human Ethics Committee (IHEC ref no: IHEC/AIIMS-GKP/BMR/123/2023).
